# Effects of noninvasive neuromodulation targeting the spinal cord on early learning of force control by the digits

**DOI:** 10.1111/cns.14561

**Published:** 2024-02-08

**Authors:** Michael A. Urbin, Charley W. Lafe, Manuel E. Bautista, George F. Wittenberg, Tyler W. Simpson

**Affiliations:** ^1^ Human Engineering Research Laboratories, VA RR&D Center of Excellence VA Pittsburgh Healthcare System Pittsburgh Pennsylvania USA; ^2^ Department of Neurology University of Pittsburgh Pittsburgh Pennsylvania USA; ^3^ Rehabilitation Neural Engineering Laboratories, Department of Physical Medicine & Rehabilitation University of Pittsburgh Pittsburgh Pennsylvania USA

**Keywords:** corticospinal tract, motor control, neuromodulation, noninvasive stimulation, precision grip

## Abstract

**Aims:**

Control of finger forces underlies our capacity for skilled hand movements acquired during development and reacquired after neurological injury. Learning force control by the digits, therefore, predicates our functional independence. Noninvasive neuromodulation targeting synapses that link corticospinal neurons onto the final common pathway via spike‐timing‐dependent mechanisms can alter distal limb motor output on a transient basis, yet these effects appear subject to individual differences. Here, we investigated how this form of noninvasive neuromodulation interacts with task repetition to influence early learning of force control during precision grip.

**Methods:**

The unique effects of neuromodulation, task repetition, and neuromodulation coinciding with task repetition were tested in three separate conditions using a within‐subject, cross‐over design (*n* = 23).

**Results:**

We found that synchronizing depolarization events within milliseconds of stabilizing precision grip accelerated learning but only after accounting for individual differences through inclusion of subjects who showed upregulated corticospinal excitability at 2 of 3 time points following conditioning stimulation (*n* = 19).

**Conclusions:**

Our findings provide insights into how the state of the corticospinal system can be leveraged to drive early motor skill learning, further emphasizing individual differences in the response to noninvasive neuromodulation. We interpret these findings in the context of biological mechanisms underlying the observed effects and implications for emerging therapeutic applications.

## Graphical abstract

Sequencing depolorization events in the spinal cord via noninvasive stimulation has been shown to enhance motor output, offering possible therapeutic potential after neurological injury. Our findings show that this neuromodulation strategy enhances learning of force control underlying finger movements but depends on the physiological state of the intact corticospinal system and its responsiveness to neuromodulation.

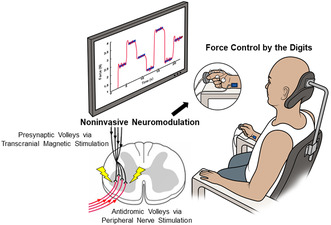

## 1 INTRODUCTION

Motor skill acquisition typically requires a large volume of task repetition performed over the course of days to years, but it is well established that the central nervous system adapts very early on (i.e., first 100 repetitions) to support learning. Animal and human studies have shown that initial performance gains are associated with altered synaptic efficacy of circuits in primary motor cortex (M1).^1^, ^2^ Widespread activation of other (sub)cortical regions is also observed due to heightened demand for attentional control at this stage of learning, leading to plasticity within cortico‐cortical networks and cortico‐subcortical loops.^3^ Neural reorganization during early motor skill learning enables coordinated activity across distributed brain regions.^4^ However, relatively less is known about adaptations in spinal circuits that integrate somatosensory input from the periphery with descending motor commands.

The spinal cord contains the necessary circuitry for rudimentary, reflexive behavior that supraspinal centers can modulate to allow more complex, differentiated patterns of distal limb control. Although traditionally viewed as a passive conduit in this regard, there is evidence of adaptation in the spinal cord early during skill learning. Spinal reflexes are suppressed on the order of minutes following novel movements^5^, ^6^ and remain down‐regulated on a more enduring basis in elite‐level performers.^7^ These adaptations are not exclusive of supraspinal input, however, as concomitant changes in presynaptic control and corticospinal excitability are also observed.^8^ In fact, corticospinal transection abolishes operant down‐conditioning of H‐reflexes in rats.^9^, ^10^ More recent work in humans has shown an uncoupling of functional connectivity between sensorimotor cortex and the cervical spinal cord during early learning of finger movement sequences, suggesting distinct contributions to skill acquisition at each level of the neuraxis.^11^ It is unlikely that these changes are mutually exclusive, however, as circuitries connecting cortical and spinal motor neurons tend to be highly integrated, particularly for distal limb muscles under monosynaptic control.^12^

Neuromodulation induces long‐term potentiation of monosynaptic contacts in the cervical spinal cord of anesthetized rats,^13^ with repeated application over a period of days producing longer‐lasting effects.^14^ Noninvasive neuromodulation targeting the spinal cord has been shown to enhance motor output of the distal limb in individuals with and without neurological injury.^15^, ^16^ These effects are elicited by repeatedly activating cortical motor neurons in M1 via transcranial magnetic stimulation (TMS) and pairing the resulting presynaptic volleys with antidromic activation of spinal motor neurons via transcutaneous stimulation of a peripheral nerve. Effects are thought to involve principles of spike‐timing‐dependent plasticity (STDP),^17^ which form the biological basis for learning. Increased size of motor‐evoked potentials (MEPs) that are elicited at a subcortical level in absence of changes in the intrinsic excitability of spinal motor neurons is thought to be mediated by STDP‐like mechanisms in synapses connecting upper motor neurons in cortex with lower motor neurons in the spinal cord. Therapeutic enhancement of activity‐based therapy by way of this paired corticospinal‐motor neuronal stimulation (PCMS) protocol has been shown in individuals with longstanding motor impairment due to spinal cord injury (SCI).^18^, ^19^

PCMS is typically administerd with the targeted muscle at rest, but individual differences in individuals with and without SCI have been observed in its aftereffects.^20^, ^21^ Administering PCMS against the background of isometric contractions upregulates corticospinal excitability in subjects who otherise do not show this effect and further enhances it in those who already show increased excitability.^21^ One consideration as it pertains motor skill (re)acquisition is how neuromodulation targeting synapses linking descending motor commands onto the final common pathway might interact with task repetition to influence learning. Distinct patterns of temporal coding by corticospinal neurons underlie acquisition of task‐specific limb movements in rodent models,^22^ and invasive neuromodulatory strategies shown to enhance re‐learning of functions compromised by neurological injury involve plasticity mechanisms unique to circuits engaged during task‐specific behavior.^23^ It therefore stands to reason that administering PCMS against the backdrop of task‐specific activity may have some effect on early motor skill learning in humans; at least in those who show an upregulation of corticospinal transmission with the same dose of stimulation. Whether and how such an effect might be achieved is unknown, but features of the task to be learned may provide some guidance.

Skilled hand movements acquired during normal development or reacquired after neurological injury involve learning how to regulate forces applied by the digits. Precision grip, for instance, requires both dynamic and static control of motor output^24^ and the ability to transition between both modes of control.^25^ While dynamic control is needed to concentrically or eccentrically grade muscle tension in order to produce the appropriate level of force, static control is needed to stabilize muscle tension in order to maintain the object between the tactile pads of the digits. Stabilizing force between transitory states, therefore, underlies our ability to perform everyday tasks such as feeding, grooming, writing, etc. Follow‐up analyses of data acquired as part of experiments from our group show that stabilizing force (root‐mean‐square error, RMSE) from an eccentrically preloaded state during precision grip is uniquely challenging.^26^ We therefore tested the effects of PCMS only, task repetition only, and PCMS coinciding with task repetition on precision grip stability from an eccentric preload. We hypothesized that synchronizing the temporally controlled deplorization sequence in the spinal cord within milliseconds of stabilizing precision grip would accelerate early learning of force control by the digits but only in those subject who show upregulated corticospinal excitability in response to conditioning stimulation.

## 2 METHODS

### 2.1 Subjects

Twenty‐three neurologically intact adults (58.3 ± 10.7 years, *n* = 12 female) underwent four testing sessions, resulting in a total of 92 sessions across all subjects. Subjects were tested under each condition in a single session without any incidence of technical failures or subject‐related issues that would have required retesting. All but one subject was right‐hand dominant as determined by the Edinburgh Handedness Inventory.^27^ All subjects were free of contraindications to noninvasive stimulation and musculoskeletal or visual impairments that would have precluded them from performing the task to be learned in these experiments. Each subject provided signed informed consent to undergo research procedures approved by the Institutional Review Board at VA Pittsburgh Healthcare System in accordance with guidelines established by the Declaration of Helsinki.

### 2.2 Experimental design

Experiments were conducted using a within‐subject, repeated‐measures design involving separate testing sessions under three counterbalanced conditions that were randomized within a subject and pseudorandomized across subjects: Rest, Sham, and Active. During conditioning stimulation, single‐pulse TMS was used to depolarize corticospinal terminals and electrical current as applied to the ulnar nerve to depolarize spinal motor neurons antidromically. Depolarization of corticospinal terminals and spinal motor neurons occurred with hand muscles at rest in the Rest condition. Depolarization of *only* spinal motor neurons occurred immediately after precision grip force was stabilized in the Sham condition. Depolarization of corticospinal terminals and spinal motor neurons occurred immediately after precision grip force was stabilized in the Active condition. In this way, comparisons of PCMS alone, task repetition alone, and PCMS during task repetition was possible. RMSE was measured at baseline and following conditioning stimulation under each condition to quantify force stability. To examine the unique effect of each condition on early learning, the number of repetitions (*n* = 24) across conditions was restricted to be well within the range of repetitions that has been used to study the early phase of learning finger movements.^28^

Testing under each condition occurred at approximately the same time each day to account for diurnal variation and was separated by at least 5 days. To establish the response of the corticospinal system to PCMS, subjects were brought back in for a fourth testing session to measure changes in corticospinal excitability and the intrinsic excitability of spinal motor neurons. Corticospinal excitability was quantified by calculating the peak‐to‐peak amplitude of MEPs elicited by TMS, and the intrinsic excitability of spinal motor neurons was quantified by calculating F‐wave peak‐to‐peak amplitude and persistence.

### 2.3 Visuomotor task paradigm

**FIGURE 1:**
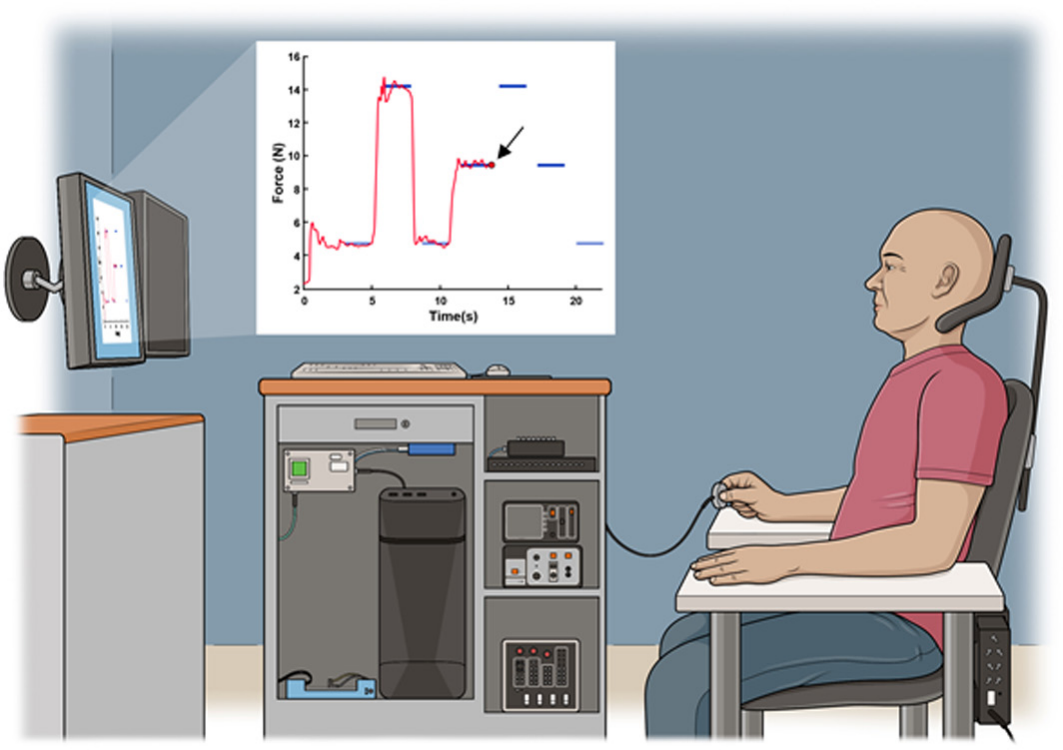
Subjects performed 6 trials on the visuomotor task paradigm before and after conditioning stimulation. The inset shows a representative target template from the display window. The goal was to control the path of the red circular cursor through the center of each target by grading and stabilizing force applied to the sensor. Target crossings at the 14% MVC target force (middle height) were grouped according to force preload: concentric = 14% MVC target force crossings preceded by the 7% MVC target force (lower height); eccentric = 14% MVC target force crossings preceded by the 21% MVC target force (upper height). Horizontal width of targets was fixed and vertical width was scaled to the variability exhibited by individual subjects during the calibration procedure.

Subjects sat in a chair approximately 1.16 m in front of a computer monitor (36.5 cm height × 61.4 cm width, 3840 horizontal pixel × 2160 vertical pixel resolution, 144 Hz refresh rate), resulting in a ~17.9° vertical × ~29.7° viewing angle. Force signals were sampled at 200 Hz using a 6‐axis force sensor (Mini40, ATI Industrial Automation). The pixel‐to‐Newton ratio was set at 64 pixels/N such that the visual indicator for force would move 64 pixels higher on the vertical axis of the monitor for every Newton of force applied along the z‐axis of the sensor. The forearm was maintained in a neutral position on an arm tray, and the sensor was held between the index finger and thumb with remaining digits fully flexed (Figure 1). Colored tape was placed on the sensor to indicate which side to place the index finger and thumb. Care was taken to ensure subjects gripped the sensor and maintained forearm position the same across all time points and conditions.^29^

A calibration procedure and visuomotor task paradigm was created in MATLAB version 2021a (The MathWorks, Inc) (see Lafe et al. 2023 for validation experiments).^26^ The calibration procedure was administered at the start of each testing condition as a warm up and to familiarize subjects with holding the sensor and stabilizing force. Measurements obtained from the calibration procedure under the first condition presented to a given subject was used to establish target templates that remained constant across all time points and conditions. The first step of the calibration procedure defined the noise floor of the sensor by sampling data while it was positioned on a stable surface. Next, subjects grasped the sensor and were verbally exhorted by the experimenter to produce a true maximum voluntary contraction (MVC). Precision grip MVC was recorded over 5 s and taken as the mean value calculated from 2 s centered around the peak force. Subjects then performed a single attempt at matching forces corresponding to 10%, 20%, 30%, 40%, and 50% of their MVC over 5 s. The display window showed a horizontal line at the force level to be matched while subjects attempted to stabilize force represented by the height of a second, horizontal line that corresponded to the force applied to the sensor. After a brief rest period, the calibration procedure was repeated a second time. Measurements obtained from this second calibration procedure were used to establish force values (vertical positions) and accuracy tolerances (vertical widths) for the set of targets displayed in a visuomotor task paradigm. The visuomotor task required subjects to increase or decrease the amount of force applied on the sensor to control a circular red cursor (10‐pixel diameter) in an upward or downward direction, respectively, as it moved across the display window at a constant rate. The objective was to control dynamic and static contractions of intrinsic hand muscles to align the path of the cursor through the center of the target forces represented by blue rectangles. Target force values were set at 7%, 14%, and 21% of MVC to approximate the low‐level forces used during precision grip and also to ensure that the maximum difference between lower and upper force targets for subjects with large MVCs did not exceed viewing limits on the display window. Vertical width of the target at each respective target force was set by regressing variability in the force signal (i.e., 1 standard deviation) from the final 2 s of recordings (i.e., where the error between the mean force and target force was minimized) onto each of the 5 force levels matched, with the intercept constrained to 0.

Subjects performed a single set of 6 trials at each time point on either side of conditioning stimulation. A total of 7 target forces were presented simultaneously at the start of each trial. The red circle moved across the display window at a constant rate, resulting in a 22‐s data sweep. The first target force was positioned 3 s into the sweep, allowing the subject adequate time to ramp force to its height. The interval between each set of adjacent targets (0.833 s) was a function of trial length (22 s), horizontal target length (2 s), and time of the initial target (3 s). This configuration was selected to approximate natural, dexterous actions that require force stability between transitory states while allowing a sufficient number of data points during each target crossing to quantify stability. The order of target force levels was pseudorandomized to ensure that (a) each force level was preceded by a different force level, resulting in 6 possible force level‐to‐force level transitions with each transition occurring only once within a trial; (b) each trial began and ended at the same force level to result in 3 target crossings at a single force level and 2 target crossings at each of the other force levels; and (c) the initial force level changed systematically such that each force level was presented as the initial target only once in a series of 3 trials.

### 2.4 Task repetition during conditioning stimulation

**FIGURE 2:**
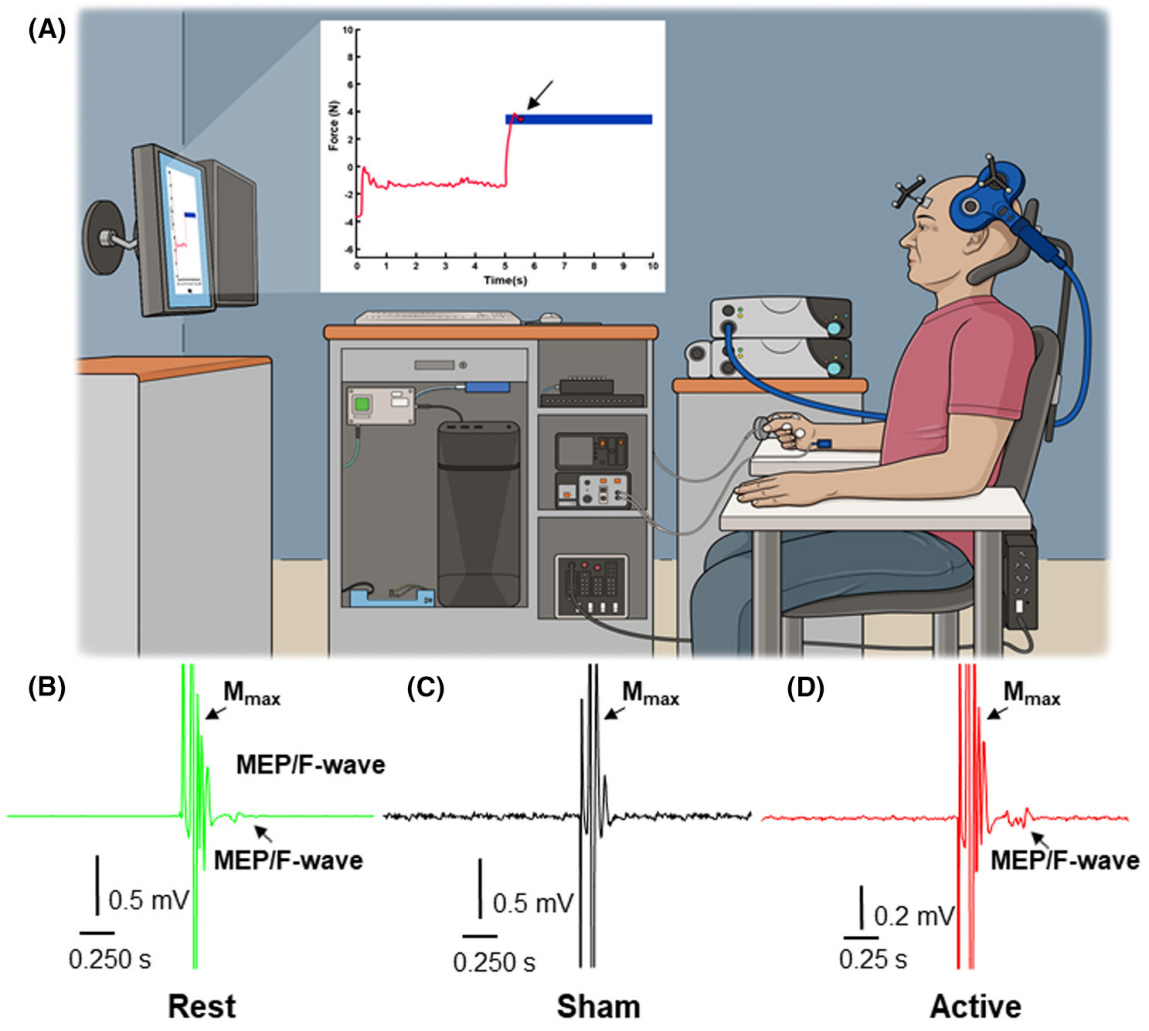
(A) Visuomotor task paradigm during conditioning stimulation in the Active condition. Note that under the Sham condition, the stimulating coil was rotated away from the optimal site during conditioning stimulation, but the perception of stimulation and task was the same. EMG recordings from a representative subject undergoing (B) Rest, (C) Sham, and (D) Active conditioning stimulation.

The forearm was maintained in a neutral position during conditioning stimulation, but the sensor was held between the index finger and thumb only in Sham and Active conditions. A single force target with a horizontal length of 5 s and height of 10% MVC was positioned in the final 5 s of a 10 s sweep (Figure 2A). The subject was instructed to begin ramping force when the red circle crossed 4 s and to maintain it on the center of the target until a stim pulse was felt on the wrist. A function built into the visuomotor task paradigm detected when the circle was maintained within target boundaries for 500 ms and sent out a TTL pulse to a data acquisition device (USB‐6001, National Instruments Corp.) connected to a separate data acquisition unit (Power1401‐3, Cambridge Electronic Design Ltd., Cambridge, UK) that generated additional TTL pulses to trigger the appropriate stimulator(s) in each condition. To avoid disparities in the time needed to complete conditioning stimulation and the number of repetitions performed as a consequence of not meeting the criterion duration for force stability, constraints on vertical target width were reduced to 10 standard deviations as computed from the calibration procedure.

### 2.5 Electrophysiological recordings

EMG was recorded from the first dorsal interosseous (FDI) muscle through surface electrodes (Ag‐AgCl, 10 mm diameter) secured to the skin in a muscle belly‐tendon montage. The FDI muscle was selected because it is involved in precision grip,^30^, ^31^ generating a flexion force about the metacarpophalangeal joint.^32^ The skin was prepared with an abrasive cream and cleansed with alcohol prior to electrode placement. Signals were amplified, band‐pass filtered (200–2000 Hz), and sampled at 2 kHz (Power1401, *Signal*, Cambridge Electronic Design Ltd., Cambridge, UK). A 200 Hz low‐pass filter was used to aid in more accurately determining response latencies^33^ needed to depolarize corticospinal terminals and spinal motor neurons in the appropriate order and interval during PCMS.

### 2.6 Transcranial magnetic stimulation (TMS)

Single monophasic, magnetic pulses intended to elicit posterior‐to‐anterior currents in M1 were applied to the scalp by a Magstim 200^2^ stimulator (The Magstim Company Ltd., Whitland, UK) through a figure‐of‐eight coil (70‐mm loop diameter, D70). The 10–20 system was used to establish scalp locations corresponding to the vertex (Cz) and 7 cm lateral of Cz in line with the tragus of the ear contralateral to the target muscle. The optimal scalp site was determined by administering TMS pulses 7 cm lateral of Cz and moving the coil in ~1‐cm increments in anterior–posterior/medial‐lateral directions. The coil was rotated to identify the optimal angle relative to the mid‐sagittal plan. The coil location and orientation that produced stable motor‐evoked potentials (MEPs) at the lowest possible stimulator output was set as the optimal site and recorded by a frameless, stereotaxic neuronavigation system (*Brainsight*, Rogue Research Inc., Montreal, Quebec, Canada). Coil location and orientation was monitored by the experimenter throughout the duration of each experiment using the neuronavigation system. Resting motor threshold (RMT) was obtained in each condition and taken as the minimal stimulator output needed to produce MEPs that were >50 μV in amplitude from 5 of 10 TMS pulses.^34^

### 2.7 Transcutaneous electrical stimulation

Electrical stimulation was applied to the skin overlying the ulnar nerve just proximal to the ulnar styloid at the wrist. Bipolar felt pad electrodes soaked in a saline solution were secured to the wrist with the cathode and anode separated by 2 cm and the cathode positioned opposite the FDI muscle. Foam cushions were placed under the hand and forearm to elevate both from the arm tray and avoid changes in pressure on the stimulating electrode. Electrical current (200 μs pulse duration) was supplied by a constant‐current stimulator (DS7R, Digitimer Ltd.). Current was graded higher until the peak‐to‐peak amplitude of the M‐wave saturated and, then, decreased in small increments to established M_MAX_ threshold.

### 2.8 Procedure

Following the visuomotor task calibration procedure previously described, surface EMG electrodes were attached and the subject was registered in the neuronavigation system. After establishing RMT, 20 TMS pulses (0.25 Hz) were administered at 1.2xRMT with the FDI muscle in a pre‐contracted state. Real‐time feedback of the rectified, smoothed EMG signal was displayed, and the subject was instructed to maintain sufficient tension in the muscle to hold the EMG signal on a horizontal line corresponding to ~10% to 30% of their maximum. Rectified waveforms were inspected to determine MEP latency. Then, instructions for performing the visuomotor task were given prior to the subject completing a total of 6 trials. Subjects were advised that the task was similar to the calibration procedure in that the goal would be to grade and stabilize force applied to the sensor. They were informed that target forces would be displayed as blue rectangles at three different heights, and the force they applied would be represented by a red circle that would move across the screen from left‐to‐right at a fixed speed. They were shown the red circle in advance of beginning the first trial and instructed to grade and maintain force so that it moved through the center of each blue rectangle. No qualitative or quantitative feedback was provided following each trial.

Next, another 2–3 sets of 20 TMS pulses (0.25 Hz) were administered at 1.2xRMT with the FDI muscle at rest. MEP latencies from pre‐contracted and resting states were evaluated while subjects were given a brief break in preparation for remaining still throughout the subsequent 15–20 min of the experimental protocol. After securing the stimulating electrode to the skin overlying the ulnar nerve, 40 pulses were administered (1 Hz) with current amplitude set to M_MAX_ threshold to antidromically activate spinal motor neurons. M‐ and F‐wave latencies were determined. All response latencies were entered into equations to calculate central (CCT) and peripheral conduction times (PCT) as follows:

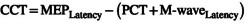




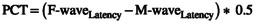




Conduction times were calculated to inform when each stimulation device should be triggered to ensure the appropriate timing of depolarization events in each subject. Stimulating devices were triggered (0.1 Hz) so that corticospinal volleys arrived in the spinal cord ~3 ms before antidromic volleys,^15^, ^16^ resulting in 100 pairs over 1000 s. TMS intensity was set at 1.5xRMT and current amplitude was set at M_MAX_ threshold for conditioning stimulation.^35^ The TMS coil was placed on its side over the optimal site in the Sham condition (Figure 2C), producing the same pressure on the scalp and the same sound from the Magstim 200^2^ as in Rest (Figure 2B) and Active (Figure 2D) conditions. After 100 paired pulses were administered, the stimulating electrode was removed from the wrist, and a brief break was provided while the visuomotor task paradigm was loaded. Subjects performed 6 trials on the visuomotor task approximately 10, 20, and 30 minutes following conditioning stimulation, resulting in 4 total time points under each condition: baseline, 10, 20, and 30 min.

Following completion of testing in all three conditions, subjects underwent testing in a fourth testing session wherein MEPs and F‐waves were recorded before and after conditioning stimulation with the FDI muscle at rest. The purpose of this experiment was to establish the response of the corticospinal system to PCMS. RMT was determined, then 5 TMS pulses were administered at stimulator outputs below and above RMT in 5‐increment steps until maximum stimulator output was reached. MEP_MAX_ was taken as the highest mean peak‐to‐peak amplitude obtained at any stimulator output. Response latencies were determined as previously described. Two sets of 20 MEPs were recorded at baseline using a stimulator output that produced a mean peak‐to‐peak amplitude of approximately 50% MEP_MAX_ to ensure similar recruitment across subjects. A single set of 20 MEPs was acquired using the same stimulator output 5, 10, 15, 20, 25, and 30 min following conditioning stimulation. Subjects were instructed to relax their hand, hold their attention stable, and fixate gaze prior to each set of TMS pulses. Subjects were re‐registered in the neuronavigation system after conditioning stimulation to account for any shifts in the tracker affixed to the forehead. Although prior work has shown that PCMS does not have an effect on the intrinsic excitability of spinal motor neurons,^16^, ^35^ F‐waves were obtained immediately before and after conditioning stimulation for verification. Table 1 contains response latencies, conduction times, interstimulus intervals, RMTs, and TMS intensities used for testing stimulation broken out by condition.

**TABLE 1:** Measurements obtained under each condition, as well as the fourth testing session that served to establish the response of the corticospinal system (CS_RESPONSE_) to PCMS.

	Rest	Sham	Active	CS_RESPONSE_
M‐wave (ms)	3.6 ± 0.5	3.7 ± 0.6	3.5 ± 0.5	3.6 ± 0.6
F‐wave (ms)	27.2 ± 3.5	27.9 ± 4.0	26.8 ± 3.8	27.6 ± 2.8
MEP (ms)	22.7 ± 2.1	22.7 ± 2.1	22.5 ± 2.2	22.7 ± 2.1
PCT (ms)	11.8 ± 1.6	12.1 ± 1.8	11.6 ± 1.8	12.0 ± 1.2
CCT (ms)	7.3 ± 1.3	6.9 ± 1.2	7.4 ± 1.5	7.0 ± 0.9
ISI (ms)	1.5 ± 2.3	2.2 ± 2.5	1.3 ± 2.7	1.9 ± 1.4
RMT (%)	47.2 ± 7.3	47.7 ± 7.5	47.0 ± 7.7	47.1 ± 7.3
MVC (N)	47.5 ± 17.7	47.0 ± 14.3	46.7 ± 15.4	–
Test stimulation (%)	1.5 × RMT	1.5 × RMT	1.5 × RMT	62.9 ± 9.1

### 2.9 Data processing

Force signals were analyzed using MATLAB version 2021a. Signals from each target crossing (2‐s time series, 400 samples) were de‐trended and filtered using a fourth‐order Butterworth filter with a 12 Hz cutoff. Force stability was quantified using root‐mean‐square error (*RMSE*) to characterize variability around the center of the target force where *n* is the total number of force samples:

$${\left[\sum {\left(Τ-f_{i}\right)}^{2}/n–1\right]}^{1/2}$$

Crossings at the 14% MVC target force were extracted and grouped according to force preload. Eccentric contractions occur when a muscle is lengthened under tension.^36^ In this precision grip task, intrinsic hand muscles are eccentrically loaded when transitioning from the 21% MVC target force to the 14% target force in order to maintain the sensor between the index finger and thumb. Eccentric preload, therefore, was defined as crossings at the 14% MVC target force preceded by crossings at the 21% MVC target force. Concentric preload was defined as crossings at the 14% MVC target force preceded by crossings at the 7% MVC target force. Given the previously described randomization scheme built into the visuomotor task paradigm, a total of 6 trials were extracted for each preload and at each time point under each condition. Trials were removed in the rare instance that the sensor was dropped or subjects reported that they lost grip.

MEPs recorded at each time point in the final testing session were inspected to verify stability of the EMG signal 100 ms prior to onset of the stim pulse. MEPs were removed from the analysis if peak‐to‐peak amplitude of the signal in this window was ≥2 standard deviations above the mean across all time points. Remaining MEPs recorded during the two sets at baseline, as well as 5‐ and 10‐min, 15‐ and 20‐min, and 25‐ and 30‐min following conditioning stimulation, were averaged and grouped, resulting in 4 total time points under each condition: baseline, 10, 20, and 30 min. Mean MEP values obtained at each time point were normalized to MEP_MAX_ to place MEP size from each subject on the same scale. We aimed to obtain a sufficient number of MEPs (*n* = 40) to derive a relatively stable estimate of corticospinal excitability at baseline and at each time point following conditioning stimulation. Responders were defined as those subjects who exhibited a facilitation above baseline at two of three time points following conditioning stimulation. Mean peak‐to‐peak amplitude of F‐waves acquired immediately before and after conditioning stimulation was computed. F‐wave persistence was determined by calculating the percentage of stimulation pulses that elicited a F‐wave. An F‐wave was considered present if peak‐to‐peak amplitude was >20 μV above background within a 20‐ms window at the predetermined latency.^37^

### 2.10 Statistical analyses

All statistical tests were computed using SPSS version 23 (SPSS Inc, Chicago), with significance set at *p <* 0.05. A 3 (condition) × 2 (force preload) repeated‐measures ANOVA was used to test for differences in RMSE from the baseline time point under Rest, Sham, and Active conditions and from Concentric and Eccentric force preloads. Repeated‐measures ANOVA was used to test for differences in MEP size across time points for the entire subject sample and separately in a subset of subjects identified as responders to conditioning stimulation. A 4 (time point) × 3 (condition) repeated‐measures ANOVA was used to test for differences in RMSE for the entire sample and, again, separately in responders. Given robust differences in RMSE between force preloads (Concentric > Eccentric), only the Eccentric preload was entered in the 4 × 3 ANOVA to test our hypothesis and increase statistical power. Individual subject baselines were averaged across conditions in the main analysis to account for prior exposure/familiarity with the task, thereby providing a relatively equal comparison of learning under each condition. This approach was adopted after verifying that baseline RMSE values were not statistically different between conditions. Paired‐samples *t*‐tests were used to test for differences between specific time points and across conditions, as well as to test for differences in F‐wave amplitude and persistence. The difference in both RMSE and MEP size relative to baseline was taken at each time point under each condition to compute the Pearson product–moment correlation between changes in behavior and physiology. All subjects were included in correlational analyses to account for proportional change independent of responder status. Normality and homogeneity of variance assumptions were verified through visual inspection of Q–Q plots and Mauchly's test of sphericity, respectively. The Friedman test and Wilcoxon signed‐rank test were used in cases where the normality assumption was not met. Greenhouse–Geisser correction statistics were used when sphericity could not be assumed. The Bonferroni test was used to correct for multiple comparisons.

## 3 RESULTS

**FIGURE 3:**
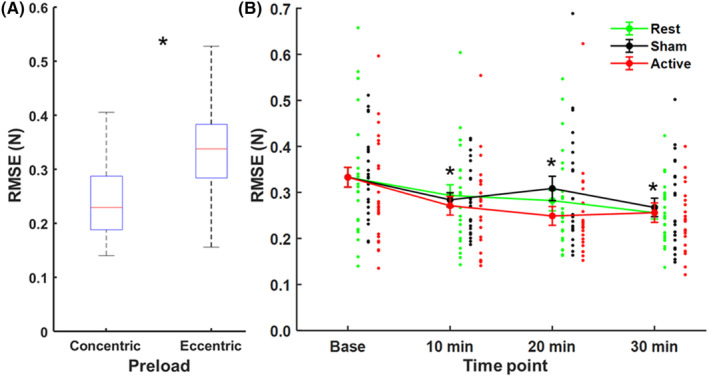
(A) Box and whisker plots showing the distribution of baselines by preload state. (B) Mean RMSE from an eccentric preload across time points under each condition for the entire sample of subjects. Error bars reflect standard error of the mean (*p < 0.05 = all conditions relative to baseline time point).

We found that subjects exhibited less force stability from the eccentric preload (M = 0.33 N, S.E. = 0.02 N) relative to the concentric preload (M = 0.24, S.E. = 0.02 N) across baseline time points without any difference between conditions. This finding was supported by a significant effect of PRELOAD (*F*_(1,22)_ = 32.02, *p* < 0.001, η_p_^2^ = 0.59) on RMSE, indicating that forces generated after lengthening contractions were less stable than those produced after shortening contractions. Mean RMSE values for the eccentric preload were relatively consistent at baseline across conditions (Rest = 0.339 ± 0.141 N; Sham = 0.359 ± 0.139 N; Active = 0.301 ± 0.115 N), ranging from 0.121 to 0.841 N across all subjects and time points. Force stability increased across time under each condition as reflected by a significant effect of TIME POINT (*F*_(3,66)_ = 14.21, *p* < 0.001, η_p_^2^ = 0.39) with reduced RMSE observed at 10 min (*p* = 0.001), 20 min (*p* = 0.003), and 30 min (*p* < 0.001) relative to baseline (Figure 3). However, there was no effect of CONDITION on RMSE. Therefore, learning occurred under each condition without any unique influence of conditioning stimulation.

**FIGURE 4:**
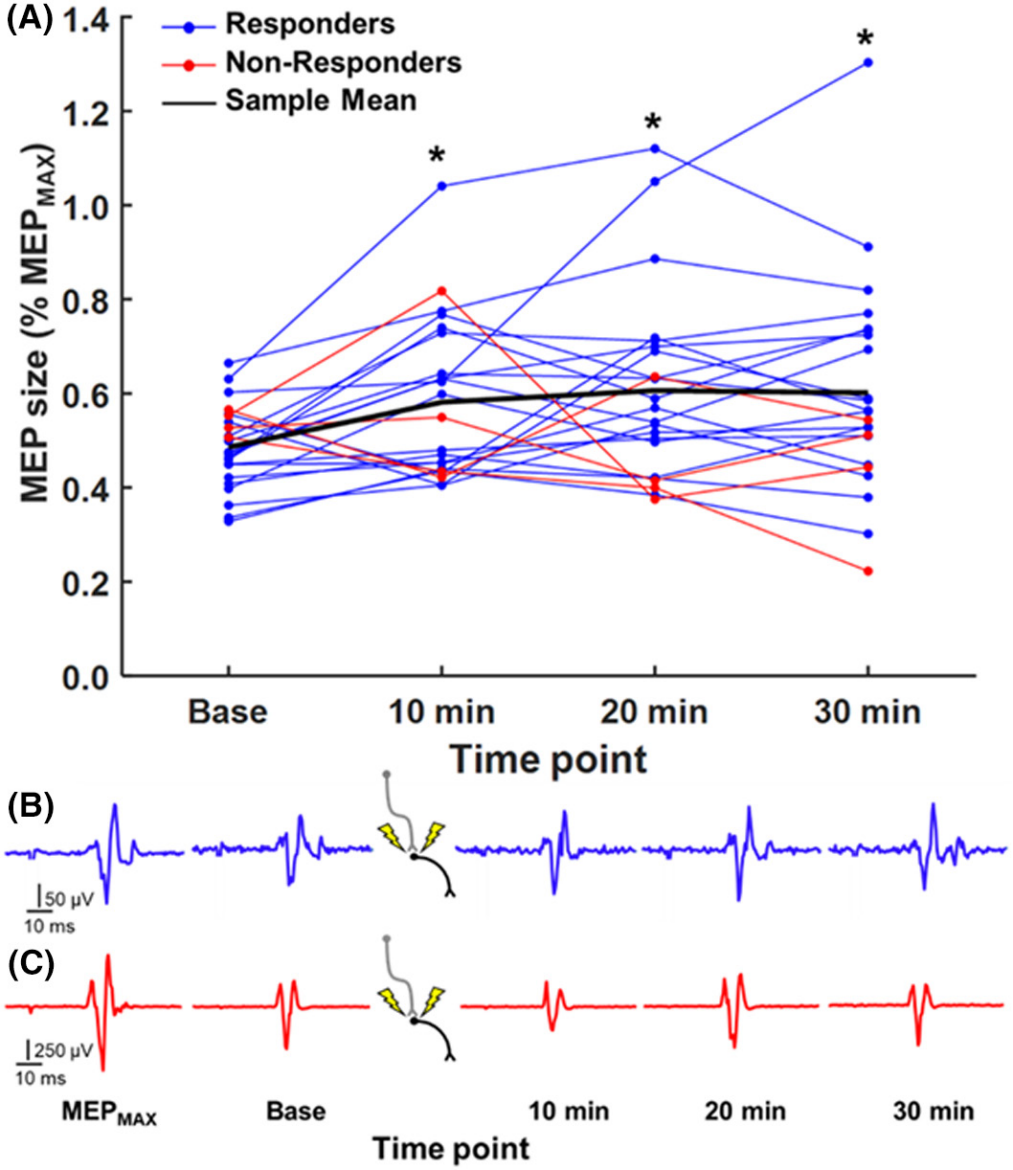
(A) MEP size across time points in response to PCMS. Blue and red lines correspond to responders and non‐responders, respectively. Black line corresponds to the mean of the entire sample (*p < 0.05 = relative to baseline time point). MEP_MAX_ and MEP at each time point in a representative (B) responder (blue trace) and (C) non‐responder (red trace).

We found that 19 of 23 subjects exhibited an upregulation of corticospinal excitability at two of three time points relative to baseline (Figure 4). MEP size normalized to MEP_MAX_ was 48.6 ± 8.9% at baseline, 58.2 ± 16.9% at 10 min, 60.6 ± 19.9% at 20 min, and 60.2 ± 22.6% at 30 min. There was an effect of TIME POINT (*X* ^2^_(3)_ = 16.096, *p* = 0.001, W = 0.23) with increased MEP size observed at 10 min (*p* = 0.008), 20 min (*p* = 0.006), and 30 min (*p* = 0.006) relative to baseline. There was no effect of TIME POINT (*Z* = 150.5, *p* = 0.704) on F‐wave amplitude from before (49.3 ± 22.9 μV) to after (50.7 ± 31.9 μV) conditioning stimulation and no effect of TIME POINT (*t*_(22)_ = 1.153, *p* = 0.261) on F‐wave persistence from before (44.9 ± 25.5%) to after (41.3 ± 23.1%) conditioning stimulation.

**FIGURE 5:**
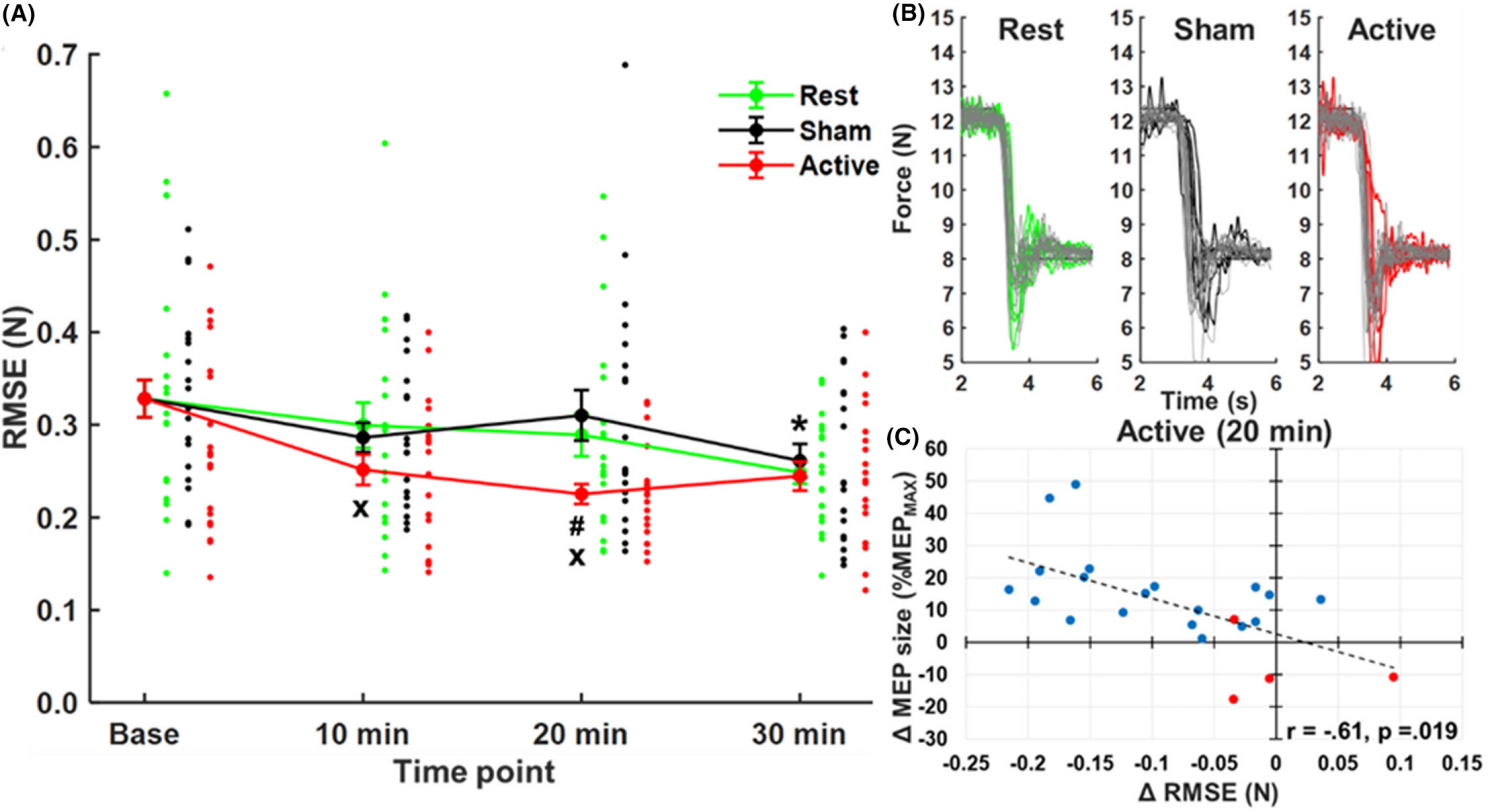
(A) Mean RMSE across time points under each condition in the subset of subjects (*n* = 19) who showed upregulated corticospinal excitability in response to PCMS at two of three time points (*p < 0.05 = all conditions relative to baseline time point, ^x^ *p* <0.05 = Active condition relative to baseline time point, ^#^ *p* < 0.05 = Active condition relative to Rest and Sham conditions). (B) Force traces depicting eccentric preload target crossings from a representative subject in each condition (i.e., 21% MVC to 14% MVC). Traces corresponding to trials from the unique baseline under each condition are color‐coded, and traces corresponding to trials across time points following conditioning stimulation are shown in gray. Note the relative stability of force traces (lower target force) following conditioning stimulation. (C) Scatter plot showing correlation between changes in RMSE and MEP size detected at the 20‐min time point under the Active condition (blue data points = responders, red data points = non‐responders).

Noting individual differences observed in response to conditioning stimulation, the 4 × 3 repeated‐measures ANOVA was reduced to include only responders, revealing a significant TIME POINT × CONDITION interaction (*F*_(3.985,71.727)_ = 2.595, *p* = 0.044, η_p_^2^ = 0.13) on RMSE (Figure 5A). Force recordings from a representative subject are shown in Figure 5B. Force stability increased at 30 min under Rest (*p* < 0.001) and Sham (*p* = 0.019) conditions relative to baseline, indicating that learning occurred under both conditions. However, force stability increased at each time point following conditioning stimulation relative to baseline under the Active condition (*p* < 0.001, all comparisons). Force stability was also significantly greater under the Active condition relative to Rest (*p* = 0.024) and Sham (0.014) conditions at 20 min following baseline, indicating that synchronizing depolarization events within milliseconds of stabilizing precision grip accelerated early learning of force control by the digits. Pearson correlation using the Benjamini‐Hochberg method to control for the false discovery rate (0.05 level) revealed a linear relationship between changes in RMSE and MEP size relative to baseline but only for the Active condition at the 20‐min time point (*r* = −0.61, *p* = 0.019, Figure 5C). There were no statistically significant correlations for the remaining time points under the Active condition (10‐min: *r* = −0.17, *p* = 0.653; 30‐min: *r* = −0.41, *p* = 0.149) and both Rest (10‐min: *r* = 0.10, *p* = 0.737; 20‐min: *r* = −0.02, *p* = 0.937; 30‐min: *r* = −0.24, *p* = 0.503) and Sham (10‐min: *r* = 0.15, *p* = 0. 653; 20‐min: *r* = −0.42, *p* = 0.149; 30‐min: *r* = −0.32, *p* = 0.303) conditions.

## 4 DISCUSSION

The purpose of these experiments was to understand whether noninvasive neuromodulation targeting the spinal cord can enhance early learning of force control by the digits. In addressing this purpose, we also sought to determine if neuromodulation interacts with task‐specific activation of the corticospinal system to influence learning. Our findings show an acceleration of early motor learning that depends on the state of the corticospinal system and its responsiveness to conditioning stimulation. Although the overall size of the effect was small, the ability to stabilize force did increase relative to baseline under each condition. There was evidence of a proportional change in physiology and behavior but, interestingly, only under the Active condition and at the same time point when force stability peaked and was greater relative to Sham and Rest conditions. We interpret these findings in the context of mechanisms underlying the observed effects and implications for emerging clinical applications on hand function after neurological injury and with advanced age.

Similar to paired‐associative stimulation (PAS) targeting cortex,^38^ PCMS targeting the spinal cord is thought to engage STDP mechanisms used to explain learing at the circuit level.^17^ STDP alters synaptic efficacy through strong depolarization events that modify N‐methyl‐D‐aspartate (NMDA) receptor channels. Accordingly, administration of an NMDA receptor antagonist abolishes PCMS aftereffects in humans.^39^ This finding coupled with several studies showing electrophysiological evidence of changes in excitability that occur at a subcortical, pre‐spinal motoneuronal level suggests that the transient effects of PCMS are mediated by STDP‐like mechanisms. How these mechanisms interact with the endogenous pattern of task‐specific activation underlying movement is unclear, but consideration of how other neuromodulatory strategies exert their aftereffects may provide some clues.

Reinforcement learning is thought to play a role in vagus nerve stimulation where success‐paired stimulation is particularly important during early motor skill learning.^23^ Learning enhancement by way of this neurobiological phenomenon appears to rely on reinforcement cues^40^ and, therefore, also would appear to be strongly tied to activity in cortical circuits. Perception of error‐based information drives exploration while learning a novel task,^41^ and varying motor output serves as a means to explore task dynamics through visual feedback.^42^, ^43^ PCMS does not have a direct effect on cortical networks involved in perception, yet we found that synchronization of the criterion behavior (i.e., force stability) with the temporally controlled sequence of depolarization events in the spinal cord enhanced learning. Since repetition on the visuomotor task engaged cortical circuitry that may be subject to reinforcement learning, one plausible explanation is that the exploration‐exploitation process^44^, ^45^ interacted with a distinct type of *reinforcement* in the spinal cord. Cortical and spinal circuits are integrated, so strengthening transmission of descending motor commands onto the final common pathway may have further enabled this interaction to occur. Alternatively, the effect may have been restricted to enhanced transmission across synapses downstream and was more or less independent of learning mechanisms in supraspinal networks. Further work is needed to better understand this interaction.

Another consideration related to this interaction is the incongruity between features of the task performed during conditioning stimulation and features probed for change. We tested for changes in force stability from an eccentrically preloaded state because performance was notably weaker and, therefore, amendable to improvement. However, conditioning stimulation took place after precision grip force was stabilized from a concentric preload with looser task constraints (i.e., wider vertical target boundaries). We did this to minimize the potential for misses that would have created a potential source of variance between subjects in the duration of the experiment and the number of task repetitions performed. Whether stricter task constraints that are specific to the deficiency in force control yield stronger gains in learning requires further study. Although the magnitude of effects needed to drive meaningful gains in function for emerging clinical applications of PCMS remain unclear, another related question is how it should be administered to maximize gains.

To date, all mechanistic and therapeutic studies have administered PCMS under the premise that it acts as a primer for upregulating the corticospinal system. In the first therapeutic trial, PCMS was administered prior to sessions of activity‐based therapy focused on impaired upper and lower limb function in individuals with SCI.^18^ Functional improvements occurred in both the intervention and sham groups, but gains in corticospinal transmission and maximum voluntary contraction were preserved 6 months after treatment only in the intervention group. In a more recent trial, a method was developed to simultaneously target multiple upper and lower limb muscles with PCMS prior to activity‐based therapy.^19^ Increased walking speed and corticospinal excitability were observed in the intervention group, and a prospective cohort that underwent twice the volume of retraining (20 vs. 40 sessions) showed gains in grasping and walking ability which were accompanied by improvement on quality‐of‐life indexes. All outcomes were stable 9 months following treatment. A recent mechanistic study in neurologically intact young adults also showed a priming effect of PCMS on subsequent learning of ballistic index finger movements.^46^ While PCMS is one of the few noninvasive means shown to enhance movement potential, other methods involving intermittent inhalation of hypoxic air^47^ or operant conditioning of spinal reflexes^48^ also show priming effects on motor output that are thought to rely on spinal adaptation.^37^ In the current study, priming in the Rest condition did not lead to the same gains in learning when compared to the Active condition, but it did result in comparable gains compared to the Sham condition which still involved task repetition. Stated another way, PCMS alone elicited gains that were similar to engaging in practice. Whether PCMS should be administered prior to or during task repetition appears to be an important question from a practical standpoint that requires further study.

Recently, there have been significant advances in the development of implantable assistive technologies (e.g., brain‐computer interface, spinal cord stimulation, etc.) capable of replacing or enhancing motor functions in people with neurological injury. Considering drawbacks inherent to implantable devices and also that some level of connectivity is often preserved between brain and spinal cord after neurological injury, noninvasive strategies that effectively engage and direct plasticity mechanisms in a way that supports function may offer a more practical solution for sizable segments of these clinical populations (e.g., spinal cord injury, stroke, etc.). This is a relevant consideration given that recovery rates are dissatisfying,^49^, ^50^ healthcare services are expensive, and the volume of retraining possible within allowable services is limited.^51^ Noninvasive forms of neuromodulation, although still early in development, may drive meaningful changes in motor function that otherwise might not be possible with activity‐based therapy alone. In general, there is a need to enhance the effects of activity‐based therapies, as improvements observed in clinical settings do not always translate into everyday life.^52^ The implication here is that neuromodulatory adjuncts working in synergy with activity‐based therapies may help rehabilitation medicine reach the threshold where improved motor function actually translates into reduced disability in everyday life.

Our visuomotor task paradigm was intended to approximate the naturally changing forces exerted on an object during everyday tasks, such as a fork during eating or a pencil during writing, while allowing sufficient experimental control to characterize force stability. Whether effects on force control observed here generalize to hand movements used in activities of daily living requires further study. Single‐joint actions underlie whole limb behaviors (e.g., reaching and grasping), and muscle weakness following neurological injury is more or less uniformly distributed across arm and hand muscles.^53^ Moreover, a minimal range of motion in specific joint actions (e.g., wrist extension) is often needed to qualify for activity‐based therapy. Noting results of prior therapeutic trials,^18^, ^19^ PCMS may enhance movement potential in specific joint actions that support whole limb behavior to improve overall motor function or enable someone to qualify for activity‐based therapy who otherwise would not. How this might be achieved during open chain movements without a force criterion such as the one used in the current study is unclear, but other measurements tools (e.g., EMG, limb kinematics, etc.) may prove useful to this end.

Finally, our findings add to growing evidence of individual differences in the response to noninvasive neuromodulation.^20^, ^21^ Which factors mediate the response in a given individual are not well understood but history of physical activity, for example, has been shown to explain the physiological response to neuromodulation targeting M1.^54^ Historical factors aside, physiological profiles tend to be rather diverse after neurological injury. Diminished hand function with advanced age in absence of neurological injury is also complex and multifactorial.^55^ Further work is needed to identify “what” factors underlie these individual differences. Nevertheless, findings from the current study provide preliminary insights into how noninvasive neuromodulation targeting the spinal cord may leverage the adaptive capacity of the nervous system and influence the rate at which early motor skill learning occurs.

### 4.1 Limitations

Several considerations related to the experimental design and interpretation of results from the current study should be noted as limitations that can inform future work. For example, there are multiple aspects of PCMS administration that warrant further scrutiny. Recent work has shown evidence of a dose‐dependent effect, with a higher number of paired stimuli (180 vs. 360) enhancing both corticospinal excitability and performance on a clinical measure of hand dexterity in SCI subjects.^56^ Another recent study showed that supramaximal current applied to the peripheral nerve produced greater changes in corticospinal excitability relative to currents above sensory threshold.^57^ The amplitude of electrical current was set to M_MAX_ threshold in the current study, which was intended to antidromically activate spinal motor neurons. Although it has been shown that F‐waves can be elicited with submaximal current,^58^ it appears plausible that the intensity of current amplitude influences the proportion of the spinal motor neuron pool recruited. Further work is needed to examine stimulation dosing and intensity to better understand how these aspects of PCMS administration influence transient changes in motor output and early motor learning.

Another consideration is how best to capture and dissociate learning from transient changes in motor output. We aimed to measure the effect of each condition on early learning and accounted for accumulated experience with the visuomotor task by averaging baselines after verifying that there was no difference in baseline performance between conditions. We further limited retention across conditions by restricting the number of repetitions performed in each session. Thus, our results reflect the effect of each condition against a generalized baseline performance. Although between‐subject experimental designs are typically used to study motor learning, a within‐subject design was chosen here, in part, to minimize systematic effects due to individual differences across subjects. Based on findings from the current study, further work using a between‐subject design and with a much larger sample is warranted.

The Sham condition in the current study was conceived of with two objectives mind. First, we aimed to administer a compelling sham that subjects could not distinguish from Rest and Active conditions. The TMS coil was placed on the scalp in all conditions. In the Sham condition, however, the coil was oriented away from cortex to avoid presynaptic depolarization but still produced the sound associated with a pulse being discharged. Given that a muscle twitch was still elicited by nerve stimulation, the only noticeable difference to subjects was whether or not they performed the task while stimulation pulses were applied (i.e., Rest vs. Sham and Active). Second, we aimed to activate spinal motor neurons antidromically as occurred in Rest and Active conditions but without the presynaptic depolarization that is thought to drive STDP‐like effects via PCMS. Although we are not aware of any previous work showing effects of 0.1 Hz peripheral nerve stimulation (100 pulses) on force control, our sham may not be a true sham control.

Finally, when to acquire MEP data and how to establish responder status in the context of these experiments are unknowns. MEPs elicited by magnetic stimulation of M1 are variable, and one source of variation that can be difficult to control is the state of the subject.^59^ We opted to take MEP measurements in a separate session after subjects completed testing under each condition because they were better able to regulate muscle relaxation and attention during stimulation procedures. We reasoned that obtaining MEP measurements after subjects were familiar with stimulation would help preserve data integrity. Acquiring both MEP and force data in the same session would appear most optimal, but this approach is complicated for a couple of reasons. First, technical considerations related to the experimental setup can make it difficult to obtain measurements on a strict time course. Second, some subjects in our sample struggled to fully relax their hand muscles, and this was further exacerbated when transitioning from performing the task. It also seems plausible that performance on the task might interact with transient changes in corticospinal excitability in ways that may not yet be fully understood. For these reasons, we opted to acquire MEP and force data in separate sessions. Although this precluded us from observing the unique effect of each condition on corticospinal excitability, it is interesting that proportional changes in behavior and physiology emerged under the same condition and at the same time point where force stability peaked at the group level.

With regard to responder status, we did not come across any established criteria for defining responders in prior PCMS studies. Due to the highly variable nature of MEP responses, defining responders based on a facilitation at a single time point seemed spurious, and facilitation at all three time points might have masked a real effect. Designating responders as those who showed a facilitation at two of three time points, therefore, appeared to be a reasonable compromise.

## FUNDING INFORMATION

This research was supported by the U.S. Department of Veterans Affairs (IK2 RX002837).

## CONFLICT OF INTEREST STATEMENT

The authors declare no competing financial interests.

## Data Availability

The data that support the findings of this study are available from the corresponding author upon reasonable request.
